# The Effect of Oral Iron Supplementation/Fortification on the Gut Microbiota in Infancy: A Systematic Review and Meta-Analysis

**DOI:** 10.3390/children11020231

**Published:** 2024-02-10

**Authors:** Theoni Karamantziani, Abraham Pouliakis, Theodoros Xanthos, Konstantinos Ekmektzoglou, Styliani Paliatsiou, Rozeta Sokou, Nicoletta Iacovidou

**Affiliations:** 1 B’ Neonatal Intensive Care Unit and Neonatal High Dependency Unit, “Aghia Sofia” General Children’s Hospital, 11527 Athens, Greece; theonikar@med.uoa.gr; 2 2nd Department of Pathology, “Attikon” University Hospital, National and Kapodistrian University of Athens, 12464 Athens, Greece; 3 School of Health Sciences, University of West Attica, 12243 Athens, Greece; txanthos@uniwa.gr; 4 School of Medicine, European University Cyprus, 2404 Nicosia, Cyprus; k.ekmektzoglou@euc.ac.cy; 5 2nd Department of Obstetrics and Gynecology, National and Kapodistrian University of Athens, Aretaieio Hospital, 11528 Athens, Greece; stpaliatsiou@med.uoa.gr; 6 Neonatal Intensive Care Unit, “Agios Panteleimon” General Hospital of Nikea, 3 D. Mantouvalou Str., Nikea, 18454 Piraeus, Greece; rosesok@med.uoa.gr; 7 Neonatal Department, Aretaieio Hospital, National and Kapodistrian University of Athens, 11528 Athens, Greece; niakobid@med.uoa.gr

**Keywords:** iron, microbiome, gut microbiota, systematic review, meta-analysis, neonates

## Abstract

(1) Background: Iron is an essential metal for the proper growth and neurodevelopment of infants. To prevent and treat iron deficiency, iron supplementation or fortification is often required. It has been shown, though, that it affects the synthesis of gut microbiota. (2) Methods: This paper is a systematic review and meta-analysis of the effect of oral iron supplementation/fortification on the gut microbiota in infancy. Studies in healthy neonates and infants who received per os iron with existing data on gut microbiota were included. Three databases were searched: PUBMED, Scopus, and Google Scholar. Randomized controlled trials (RCTs) were included. Quality appraisal was assessed using the ROB2Tool. (3) Results: A total of six RCTs met inclusion criteria for a systematic review, and four of them were included in the meta-analysis using both the fixed and random effects methods. Our results showed that there is very good heterogeneity in the iron group (I^2^ = 62%), and excellent heterogeneity in the non-iron group (I^2^ = 98%). According to the meta-analysis outcomes, there is a 10.3% (95% CI: −15.0–−5.55%) reduction in the bifidobacteria population in the iron group and a −2.96% reduction for the non-iron group. There is a confirmed difference (*p* = 0.02) in the aggregated outcomes between iron and non-iron supplement, indicative that the bifidobacteria population is reduced when iron supplementation is given (total reduction 6.37%, 95%CI: 10.16–25.8%). (4) Conclusions: The abundance of bifidobacteria decreases when iron supplementation or fortification is given to infants.

## 1. Introduction

Iron is essential for the growth and development of the human body [1,2,3,4]. It is involved in various biological processes due to its ability to act as an electron receptor or electron donor [1,2,3,5,6,7].

During life, the human body has different requirements for iron relative to the developmental phase at that given period [1,3,8]. The increase in iron demands cannot always be met by dietary iron; thus, oral iron supplementation and fortification are often required [5,9,10,11,12]. Ensuring iron adequacy is critical, as studies showed that iron deficiency anemia in infancy is associated with neurodevelopmental and cognitive delay [1,3,13,14,15,16,17,18], which persists despite restoring optimal iron levels [17,18,19].

The World Health Organization (WHO) recommends the administration of supplementary iron in both infants and toddlers (aged 6–23 months) to prevent/treat iron deficiency and iron deficiency anemia, especially if they live in areas where the incidence of anemia is greater than 40% [20]. A daily dose of 10–12.5 mg for 3 months of elemental iron is the recommended regimen and approach [20].

However, supplementary iron is poorly absorbed by the intestinal cells, and thus, the greatest portion of it ends up in the lumen of the colon, where it becomes available to the pathogenic gut bacteria [9,11,13,18,21,22]. Iron is an integral part of their survival, and they have developed mechanisms to bind it to ensure adequacy, whereas beneficial bacteria have limited or no need for iron [5,23,24]. Consequently, this may lead to oxidative stress, gut inflammation, and bacterial dysbiosis [3,10,18,25]. This has adverse effects, especially for infants, since their iron requirements are higher during this developmental period and their immune system is still immature, making them vulnerable to infections from iron-stealing pathogens [1,26].

Therefore, the aim of this systematic review and meta-analysis is to determine the effect of *per os* iron supplementation and fortification on the gut microbiota in infancy.

## 2. Materials and Methods

### 2.1. Search Strategy

This study is compliant with the Preferred Reporting Items for Systematic Reviews and Meta-analyses (PRISMA) guideline [27]. It is a systematic review and meta-analysis that investigates the relevant randomized clinical trials (RCTs) that determine the effect of oral iron supplementation on infantile gut microbiota and presents the cumulative results. The search for eligible studies was based on the PICO (Participants, Interventions, Comparators, Outcomes) paradigm [27]. A systematic literature search was conducted in the following databases: PUBMED, Google Scholar, and Scopus. There was no limit considering the date of publication of the studies. The Medical Subject Headings (MeSH) database was used to identify synonyms. The full search strategy used for PubMed was as follows: ((infant*[Title/Abstract] or infant*[MeSH Terms]) OR (neonat*[Title/Abstract] or neonat*[MeSH Terms]) OR (newborn[Title/Abstract] or newborn[MeSH Terms]) OR (perinat*[Title/Abstract] or perinat*[MeSH Terms])) AND ((iron[Title/Abstract] or iron[MeSH Terms]) OR (ferrum[Title/Abstract] or ferrum[MeSH Terms]) OR (Fe[Title/Abstract] or Fe[MeSH Terms]) OR (supplement*[Title/Abstract] or supplement*[MeSH Terms])) AND ((microb*[Title/Abstract] or microb*[MeSH Terms]) OR (gut[Title/Abstract] or gut[MeSH Terms])). The search strategy was adjusted accordingly for Google Scholar and Scopus. In addition, articles that were not written in English were excluded. Screening of the reference lists of selected studies was carried out in order to find RCTs that were not retrieved using the methods mentioned above (snowball search method). Then, all retrieved studies were merged into one list, duplicates were excluded, and each article was eligible for evaluation.

### 2.2. Inclusion and Exclusion Criteria

The inclusion criteria were as follows: human studies performed in healthy neonates and infants (until the age of 12 months), oral iron in the form of supplementation or fortified food, a control group, data about the gut microbiota, and articles written in English. The exclusion criteria were as follows: age of participants above 12 months, neonates that require hospitalization, studies performed in animals, chronic disease (e.g., HIV, chronic kidney failure), studies that do not provide data on gut microbiota, and articles not written in English. Screening and review were performed independently by two of the authors (T.K. and S.P), and in cases of discrepancies, a third author (R.S) was involved.

### 2.3. Data Extraction

The data extraction was performed by two independent researchers (N.I and T.K.). The final data extracted were as follows: general characteristics of the study, study design, characteristics of the groups, type of intervention (iron or no iron), and data on gut microbiota (type of bacteria and classification as beneficial or potentially pathogenic bacteria). The outcomes that were extracted include changes in gut microbiota composition from alterations in the relative abundance of the two groups of bacteria after they were classified as beneficial or potentially harmful.

### 2.4. Risk of Bias and Study Quality Appraisal

All studies fulfilling the above criteria were evaluated for risk of bias using appropriate tools (https://www.riskofbias.info/, accessed on 20 July 2023) in accordance with their type of study. Specifically, the RCTs that were included in our study were evaluated using the Revised Cochrane risk-of-bias tool (Rob2 Tool). This tool assesses five domains; the assessor must provide their judgement on each domain and on the overall bias [28]. As a result of this process, conclusions regarding the quality of each study were reached, and traffic light plots were created accordingly [28].

### 2.5. Statistical Analysis

All included studies were analyzed at the level of systematic review, and the studies that had quantitative data were analyzed at the level of a meta-analysis. For each evaluated variable, a Forest Plot diagram is presented along with the relative results, and a funnel plot for the evaluation of the publication bias is also produced. The desired results occur when studies fall within the area of the triangle (funnel). In situations where some studies report results that are very different from the rest, the meta-analysis considering the specific subject has been repeated without them in an effort to improve the heterogeneity index I^2^ and τ.

Meta-analysis was based on both the fixed and random effects models. As detailed information for each individual case was not available, the analysis was based on aggregated data reported in the studies. Data were extracted from the relevant studies after determining their significance. The meta-analysis software was the R statistical computing language (edition 4.3.0) [29], under the Microsoft Windows operating system, and with the specialized meta-analysis package meta [30,31]. In studies where mean value and standard deviation (SD) were not available, the median and 1st and 3rd quartiles were used in order to estimate the mean value and the relevant SD using the method of Hozo et al. [32]. In the manuscript, maximum and minimum values were also reported, and an improved method for mean and SD proposed by Bland [33] was used. The Deep Meta Tool, Version 1, was used for these estimations [34]. In cases that only minimum and maximum values were reported, the range rule was used to estimate the SD, whereas the mean value was used as the median. For studies reporting case series with detailed information for each patient, the mean value and SD were calculated by the authors. Multiple groups treated with different agents reported in a single study were considered separate during the meta-analysis.

## 3. Results

**Figure 1:**
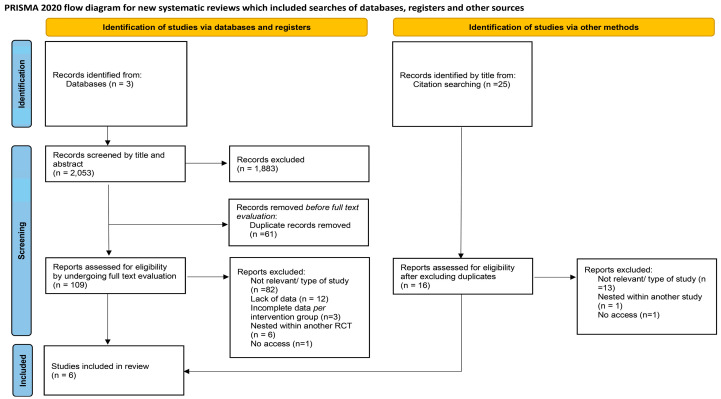
PRISMA flow chart for the study selection process and outcomes [35].

Initially, 2053 results were identified, and 1883 were excluded via title and abstract assessment. A total of 170 papers were retrieved, and 61 duplicates were excluded. The remaining 109 papers underwent full-text evaluation. Screening of the reference lists of these studies generated 16 more studies for full-text evaluation. Following this step, six studies fulfilled the pre-determined inclusion and exclusion criteria. Three studies were excluded due to incomplete data on gut microbiota per intervention group and two that were not accessible. The study selection process is presented in the following PRISMA flow chart (Figure 1).

### 3.1. Study Characteristics and Participants

Two (from the six) studies were conducted in Africa, two in Europe, one in the USA, and one in Canada. One of them was published in 1985, and the rest were published between 2013 and 2019 (Table 1). All studies were RCTs as *per* their study design. Infants were exclusively or predominately breastfed at enrollment in four of the studies. In one study, the intervention started at birth, and one study does not provide information concerning whether the infants were breastfed at enrollment. Apart from the fact that breastmilk is the best source of nutrition, prebiotics, and probiotics for neonates and infants [36,37,38,39,40], breastfeeding also minimizes the possibility of other external interventions prior to the beginning of the studies.

Regarding the method employed to determine the relative abundance of bacteria and the various taxonomic levels (such as phylum, class, order, family, and genus), the studies had different approaches, specifically (a) Mevissen-Verhage et al. [41]: incubation and identification of viable counts of bacteria; (b) Krebs et al. [42]: 16S rRNA gene amplicon sequencing of the V1V3 region, relative abundance of the microbiome data at the phyla and genera; (c) Cheung et al. [43]: 16S rRNA gene amplicon sequencing of the V4 region, normalized log-scale counts of bacterial genera; (d) Tang et al. [10]: 16S rRNA gene amplicon sequencing of the V4 region, relative abundance of the microbiome data at the phyla and the genus levels; (e) Qasem et al. [44]: 16S rRNA gene amplicon sequencing of the V3–V4 region, median relative abundance of dominant phyla and families; and (f) Simonyte Sjodin et al. [45]: 16S rRNA gene amplicon sequencing of the V3–V4 region».

### 3.2. Age

**Figure 2:**
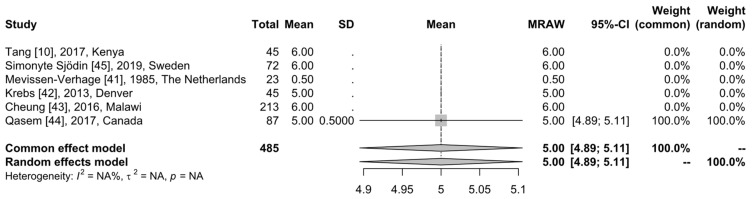
Forest plot of the number of participants and their age. First column shows the first author, the year of publication, and the country. The second column shows the number of participants. The third column refers to the mean value of their age, and the fourth column shows the standard deviation. The following three columns show the forest plot, the mean value, and the 95% confidence interval. The remaining two columns show the weights for the common and random effect models [10,41,42,43,44,45].

We only considered studies that provided data on the gut microbiome. Six studies were available for further analysis. The reported age of the participants in these studies was as follows: in three studies, 6 months; in one study, 5 months; in one study, neonates at birth; and in one study, 0–6 months. Thus, there is compatibility regarding the age of the participants. The forest plot of age is presented in Figure 2 and depicts the sample size of the studies and the age of the neonates and infants. Due to the fact that the standard deviation was not available and could be estimated in only one study, it was not possible to evaluate the age of the total population. As a result, we cannot extract homogeneity conclusions. However, there are more data available for the participants’ age for the individual study groups.

### 3.3. Groups

We searched for studies that involved at least two groups of neonates or infants: a group that received *per os* iron (in any form or in combination with any other nutrient) and a group that did not receive iron supplements (nutrition with meat is included in this group). In some studies, there was more than one group that received iron supplements; thus, such groups were combined into one.

**Figure 3:**
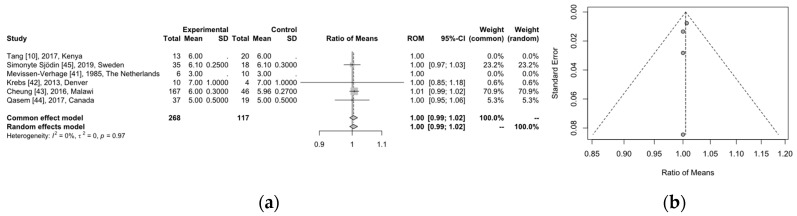
(**a**) Forest plot presenting the meta-analysis results for the age of participants per group, using both common effect and random effects models. The first column refers to the first author of the study, the year of publication, and the country of origin. The following three columns refer to the intervention groups, and they show the number of participants, the mean value of their age, and the standard deviation. Respectively, the next three columns refer to the same data for the control groups. The eighth and ninth columns show the graph for the ratio of means—ROM. The last three columns present the 95% confidence interval, the common effect model, and weight for the common and random effect models. (**b**) The relevant funnel plot for the risk of publication bias. [10,41,42,43,44,45].

Regarding the age of the infants, we did not expect differences since most of the initial studies included infants around the age of 6 months old. However, we performed the meta-analysis for the age between the two groups (one group receiving iron supplement and the other group without iron supplement) for the rate of means in order to ensure that there are no differences in the infants’ age between the two groups across the studies. The results are summarized in the forest plot below (shown in Figure 3a) with very good heterogeneity (I^2^ = 0) and an aggregated rate of means equal to one (95% CI: 0.99–1.02). This is indicative that the studies have excellent agreement for the age of their participants; therefore, age is not a factor that may affect subsequent analysis. Furthermore, the risk of bias is also limited, as all the studies fall within the funnel in the relevant funnel plot, indicative of low publication bias (shown in Figure 3b).

### 3.4. Microbiome Analysis

We separated the bacteria into two groups: (i) the beneficial bacteria, such as *Bifidobacterium* and *Lactobacillus* spp. that induce a positive effect on neonates and (ii) the potential pathogenic bacteria, whose increased population has a negative effect on neonates and infants. Following that, we investigated their population for each arm of the available studies. The results are summarized in Table 1.

Although all six of the studies report quantitative data, they do not use the same reporting methods. To be more specific, the study by Mevissen-Verhage et al. [46] reports colony-forming units (CFUs) *per* feces gram, whereas the study by Cheung et al. [43] reports log-transformed normalized read counts. The most promising meta-analysis studies were those by Tang et al. [10], Simonyte Sjodin et al. [45], Krebs et al. [42], and Qasem et al. [44], as they all report relative abundance. Therefore, we focused on the last four studies for further investigation of the reporting details since the other two studies had incompatible data.

**Figure 4:**
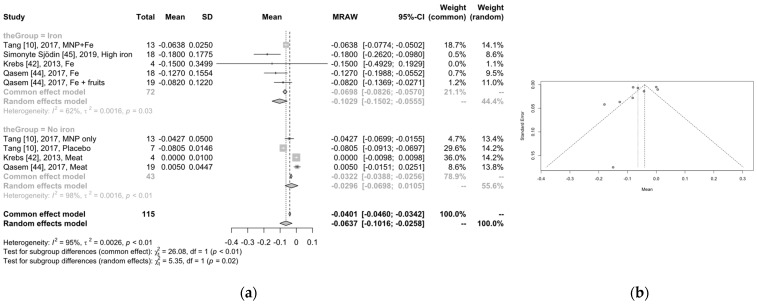
(**a**) Forest for the population of bifidobacteria per group, using both common and random effects models. The first column refers to the first author, year of publication, and group of participants (intervention or control group, respectively). The next three columns refer to the number of participants, the mean value of the changes in the bifidobacteria population, and the standard deviation. The next two columns show the mean value graph and the mean value. The last three columns present the 95% confidence interval and the common effect model and random effect model weights. At this point, there are certain things that need to be noted: 1. For the results by Simonyte Sjodin et al. [45], Krebs et al. [42], and Qasem et al. [44], the standard deviations of differences were estimated. 2. For the study by Krebs et al. [42], the arm involving Fe + Zn was excluded as the introduction of Zn could alter the microbiome. 3. For the paper by Simonyte Sjodin et al. [45] and Qasem et al. [44], the difference based on the bacteria expression before and after the intervention was calculated. 4. Different arms from the same studies were treated as providing separate results; for instance, the Fe group and the Fe + fruits group in the study by Qasem et al. [44], and the MNP only and placebo in the paper by Tang et al. [10]. 5. We applied both fixed and random effects models since we were not aware of the underlying phenomena. (**b**) The corresponding funnel plot for the risk of publication bias.

The results for the iron and non-iron groups are presented in the forest plot below (shown in Figure 4a). There is very good heterogeneity in the iron group (I^2^ = 62%) and excellent heterogeneity in the non-iron group (I^2^ = 98%). According to the meta-analysis outcomes, there is a 10.3% (95% CI: −15.0–−5.55%) reduction in the *Bifidobacteria* population when iron supplement is provided and a −2.96% reduction for the non-iron group (due to the results reported by the study of Tang). In total, there is a confirmed difference (*p* = 0.02) in the aggregated outcomes between iron and non-iron supplement, indicative that the population of *Bifidobacteria* is reduced when iron supplement is given (total reduction: 6.37%, 95% CI: 10.16–25.8%). Some studies may also introduce some publication bias, as shown in Figure 4b.

Unfortunately, the included studies report different species of potentially pathogenic bacteria, and as a result, it is not feasible to perform a meta-analysis for their population. The extracted data from the studies are presented in the Supplementary Table S1.

### 3.5. Quality of Studies

**Figure 5:**
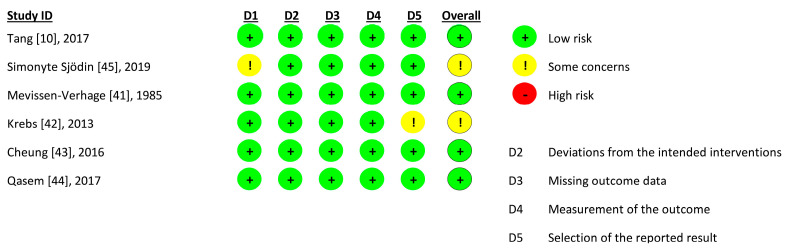
Corresponding traffic light plot for the quality appraisal of each study according to RoB2Tool [10,41,42,43,44,45].

Outcomes regarding the quality of each study accessed by the RoB 2 tool are presented in the form of a traffic light plot (shown in Figure 5). Four of the studies scored low risk in all domains, and two of them were assessed as “some concerns”. In the study by Simonyte Sjodin et al. [45], there are some concerns, as there is no information on whether the participants knew if they were in the low iron group or the high iron group. Furthermore, participants in the iron drop group probably knew the group they belonged to. In the study by Krebs et al. [42], there is no information on whether there was a pre-specified analysis plan that was used for the interpretation of the data before unblinded outcome data were available for analysis.

## 4. Discussion

This work focuses on investigating whether supplemental per os iron may affect gut microbiota in infancy in an unfavorable way by acting as a substrate for pathogenic gut bacteria.

Iron deficiency is negatively correlated to behavioral, cognitive, and motor development, as well as impaired immune responses [1,3,7,13,14,15,16,17,18]. Ensuring iron adequacy is critical, as iron deficiency anemia in infancy is associated with neurodevelopmental and cognitive delay that persists even if optimal iron levels are achieved [7,17,18]. By the age of 6 months, breastfed infants are dependent on supplementary solid foods as sources of iron [7,14,26,44,47]. In order to prevent iron deficiency, oral iron supplementation and fortification are often required [5,9,10,11,12].

Although gut microbiota and microbiome have different definitions, the confusion between these two terms has led to their indiscriminate use [9]. The development of the intestinal microbiota goes through three distinct phases of development: the developmental phase, the transitional phase, and the stable phase [1]. During the first year of life, bifidobacteria dominate (up to 90% of the total microbiome in healthy breastfed infants—the developmental phase); after the first year of life and up to age three, a steady decrease in their population occurs (the transitional phase); and after the age of three, the gut microbiota resembles that of adults (the stable phase) [21,48,49,50,51]. Both bifidobacteria and lactobacilli enhance the gut barrier by competing against other microorganisms and preventing them from colonizing the intestine [1,21,24,52,53,54,55]. Lactoferrin in breastmilk is a promoting agent for beneficial bacteria due to its high affinity for iron ions, thus making iron unavailable for pathogenic microorganisms [2,56]. However, excess iron in the gut lumen may stimulate growth and proliferation of pathogenic microbes in a possible dose-dependent manner [2,10,12,57], as the majority of intestinal microbes (with the exception of the beneficial symbiotic bacteria of the genus lactobacillus and bifidobacterium) require iron for survival and growth [2,57]. In order to acquire iron, most pathogenic bacteria produce siderophores, which are chelating molecules with high affinity for iron [2,5,23,58]. Siderophores can also bind other metals, such as manganese, which has a negative impact on the population of lactobacilli, as manganese is an essential metal for their growth and survival [21].

In clinical practice, oral supplementation begins around the age of six months of life [20]. In one of the selected studies, the intervention started at birth, and in the majority of them, iron intervention occurred at approximately 6 months of age. In our study, a meta-analysis was performed using both the fixed and random effects methods. Based on the reported age of participants in the selected studies, it was shown that there is compatibility regarding the age of participants, although homogeneity conclusions could not be extracted due to the lack of data per individual. However, the meta-analysis for the age of participants between the two groups based on data per study group showed very good heterogeneity; thus, age did not affect the subsequent analysis.

To our knowledge, the unfavorable effect of iron on gut microbiota in human infants was described for the first time in 1985 by Mevissen-Verhage et al. [41,46]. The study concluded that gut microbiota composition in infants on fortified formula milk was considered unfavorable, as it was putrefactive (presence of aerobic and anaerobic microorganisms, low presence of bifidobacteria, and a high presence of E.coli and clostridia), whereas infants on unfortified formula milk had a microbiome similar to that of breastfed infants, which appeared to enhance the organism’s resistance to colonization by potentially pathogenic microbes as bifidobacteria predominated [41,46].

In agreement with the studies by Mevissen-Verhage [41,46], Tang et al. and Simonyte Sjodin et al. observed that iron supplementation (whether in the form of iron-fortified micronutrient powders, MNPs, milk, or iron drops) leads to a decrease in bifidobacteria, which was statistically significant [10,45]. In another study from Kenya, Jaeggi et al. administered to infants MNPs containing iron (two groups of infants received a different form of iron: either 2.5 mg NaFeEDTA or 12.5 mg ferrous fumarate) and MNPs without iron [49]. Their findings showed that the iron groups showed an increase in enterobacteria (specifically Escherichia/Shigella, *p* = 0.048), an increase in the enterobacteria/bifidobacteria ratio (*p* = 0.02), and clostridium (*p* = 0.03) [49]. It was also observed that there was an increased incidence of diarrhea and intestinal inflammation in these groups [49]. The study by Paganini et al. (2017) had similar results (*p* < 0.01) [59].

Our findings are in agreement with the results of the above studies regarding the bifidobacteria population. Based on the provided data from the studies that met inclusion criteria, meta-analysis was feasible only for the changes in the abundance of bifidobacteria. Both fixed and random effects models were applied. Our meta-analysis (which included four studies that met the inclusion criteria) is in agreement with the findings of the first group of researchers. The population of bifidobacteria showed a 10.3% (95% CI: −15.0–−5.55%) reduction in the iron group and −2.96% reduction for the non-iron group (*p* = 0.02). Overall, their population is reduced when iron supplementation/fortification is given (overall reduction: 6.37%, 95% CI: 10.16–25.8%). It should be reminded that the dominance of bifidobacteria results in protecting the host by competing against pathogens for nutrients, preventing colonization, producing bacteriocins, and altering the host’s immune response [10,12,40,53]. Furthermore, it has been shown that bifidobacteria may affect the host’s mental health in a positive way through regulation of endocrine and immune mediators of the gut-brain axis, decrease the risk of developing obesity, increase bone mass density, and decrease the symptoms of atopic dermatitis and lactose intolerance [38,53]. A decrease in their population could lead to unfavorable outcomes.

In 2013, Krebs et al. studied the effect of different forms of iron on the intestinal microbiome of 5-month-old breastfed infants [42]. Although they observed a reduction of bifidobacterium and lactobacillales with a parallel increase of bacteroidales in the iron group compared to the other two groups, the researchers did not find evidence that iron fortification could increase the abundance of potentially pathogenic bacteria, nor did they observe a significant increase in the population of enterobacteria in any of the groups [42]. Their sample size was small, so their study lacked statistical power [42]. In the study by Qasem et al., researchers observed certain changes in the mean relative abundance of microorganisms after introducing three types of complementary foods (iron-fortified cereal, iron-fortified cereal and fruit, and meat), but none of them were significant when correction for multiple comparisons was applied [44]. They concluded that fortifying foods with iron may affect the gut microbiome, and further research is required [44].

In addition, iron fortification in the form of a lipid-based nutrient supplement, LNS, or a fortified corn-soy mixture does not seem to affect the gut microbiota [43,48]. Cheung et al. observed no change in the microbiome across all groups at any age (each *p* > 0.10), and the gut microbiota at the end of the intervention period was consistent with the microbiome observed in infants living in the rural areas of Malawi [43]. The study by de Goffau et al. (2022) had a similar outcome, showing that iron administration did not significantly affect the gut microbiome of Gambian infants and toddlers [60].

Although it would be of great significance to perform a meta-analysis for more bacterial species, there were major obstacles. Data across studies was inadequate to analyze for *Lactobacillus* spp., as only two studies reported data on *Lactobacillus* spp. The species of potentially pathogenic bacteria identified in the studies differed between the studies, and as a result, a comparison was not feasible. Another limiting factor was the fact that the included studies did not use the same reporting and measurement methods for quantitative data.

It is understood that the gut microbiota plays an important role in the health of the host [4,61,62,63,64,65]. It participates in the production of nutrients and vitamins, competes with pathogenic microorganisms, stimulates angiogenesis, regulates fat storage in the host, and interacts with the developing immune system [38,40,61,62,66]. Studies showed an association between the microbiome and the risk of developing allergies, infections, and possibly obesity at an older age [61,67,68]. The causal relationship between gut microbiota and the manifestation of diseases such as gastric cancer, mucosal lymphoid tissue lymphoma, inflammatory bowel disease, and necrotizing enterocolitis is a subject of ongoing studies [22,61,62,69,70,71,72,73,74].

Iron plays an important role for microorganisms [2,57]; thus, it is crucial to determine which chemical form of iron or combination of nutrients is suitable for iron supplementation and fortification, with the aim of preventing iron deficiency and preserving a favorable synthesis of the gut microbiota [75]. Animal studies and human studies have shown that iron supplements are known to have adverse effects, causing oxidative stress, gut inflammation, and bacterial dysbiosis, which can lead to increased diarrhea prevalence [25,59,70,76,77,78,79,80,81,82,83]. Some studies have reported that the combination of prebiotics (such as galacto-oligosaccharides) and iron supplements could mitigate that effect [45,59,84,85]. Although the concomitant use of prebiotics or probiotics with iron could offer a potential alternative regimen, further research is needed in order to ensure their safety and effectiveness [55,86,87,88].

There are several limitations to our study. First, a literature search was conducted in three major electronic databases (PUBMED, Scopus, and Google Scholar). Then, the list of references for selected articles was scanned for relevant studies; however, there is always a possibility that relevant studies might have been accidentally missed. Second, the included articles were only in English, and there could be relevant studies in other languages that could affect the results. Third, the full text of two possibly relevant articles was not available, thus they were excluded, and three relevant studies did not provide separate data on gut microbiota for the intervention and control groups; therefore, they were also excluded. Fourth, the number of participants was small. Another possible limitation is the fact that the form and amount of iron being administered were not the same in all studies. Finally, diet regimens differ between countries, and theoretically, they could have had an impact on the inter-subject and intra-subject variability of the gut microbiota.

## 5. Conclusions

In summary, according to the results of this meta-analysis, there is a confirmed difference (*p* = 0.02) in the aggregated outcomes between iron and non-iron supplement, which is indicative that the bifidobacteria population is reduced when iron supplementation/fortification is given (total reduction of 6.37%, 95% CI: 10.16–25.8%). Both iron deficiency and its overabundance are connected to pathological conditions. The research interest in the effect and safety of orally administered iron—either in a form suitable for food fortification or in the form of a supplement—on the microbiome remains more relevant than ever. Further studies are required to determine the chemical form and the consequent dose of iron that can be administered in order to prevent iron deficiency anemia and simultaneously not affect the beneficial bacteria population (such as bifidobacteria) in a negative way.

## Figures and Tables

**Table 1:** Details of studies included in the analysis.

First Author	Year	Country	Study Type	Intervention	Age of Participants	Duration of Intervention	N1	Changes in Beneficial Bacteria	Changes in Pathogenic Bacteria	N2	Changes in Beneficial Bacteria	Changes in Pathogenic Bacteria
**Mevissen-Verhage** [41]	1985	The Netherlands	RCT	Iron-fortified milk.	At birth	3 months	6	Mean concentration of counts of **lactobacilli:** ~10^7^ CFU/g feces. The majority of the anaerobes were bifidobacteria, with mean counts of >10^9.5^ CFU/g feces.	Mean number of ***Escherichia coli*** in bottle-fed infants with iron: 10^9^ CFU/g feces. Mean concentration of counts of **enterococci** in fecal samples of both bottle-fed groups: 10^8^ CFU/g feces. Counts of **bacteroides:** more or less constant over time; mean concentration: 10^9^ CFU/g feces. Counts of **clostridia** in both bottle-fed groups: 10^7^ CFU/g feces (most frequently isolated microorganisms among the anaerobes in bottle-fed infants supplemented with iron).	10	Mean concentration of counts of **lactobacilli:** ~10^7^ CFU/g feces. The majority of the anaerobes were **bifidobacteria,** with mean counts of >10^9.5^ CFU/g feces.	Mean number of ***Escherichia coli*** in breastfed infants: 10^8.1^ CFU/g feces. Counts of **enterococci** in fecal samples of breastfed infants (mean concentration about 10^6^ CFU/g feces). Counts of **bacteroides:** more or less constant over time; mean concentration: 10^9^ CFU/g feces. Counts of **clostridia** in breastfed infants: about 10^6.4^ CFU/g feces. Overall isolation frequency of **clostridia** in breastfed infants: 25%, (significantly lower than in both bottle-fed groups).
**Krebs** [42]	2013	Denver	RCT	Iron-fortified cereal.	5 months	Approximately 3 months	10	**Bifidobacterium** Δ% abudance in Fe + Zn: stable (−10%, 10%), in Fe group: decreased by >10%. **Lactobacillales** in Fe + Zn: stable (−10%, 10%), in Fe group: decreased by >10%.	**Bacteroidetes** Δ% abudance in Fe + Zn: decreased by >10%, in Fe group: increased by >25%. **Enterobacteriaceae:** in Fe + Zn: stable (−10%, 10%), in Fe group: decreased by >10%. **Clostridia** Δ% abudance in Fe + Zn: increased by >25%, in Fe group: stable (−10%, 10%).	4	**Bifidobacterium** Δ% abudance in meat group: stable (−10%, 10%). **Lactobacillales:** stable (−10%, 10%).	**Bacteroidetes** Δ% abudance in meat group: stable (−10%, 10%). **Enterobacteriaceae:** decreased by >10%. Clostridia Δ% abudance: increased by >25%.
**Cheung** [43]	2016	Malawi	Substudy of a four-arm RCT	Iron fortification.	6 months	12 months	167	Soya LNS seemed to have higher counts of some **lactobacillus** species. **Bifidobacteria:** decreased over time (each *p* < 0.001). Lactobacilli: relatively constant. **Bifidobacterium** log-transformed normalised read counts CBS: baseline (median: 5.2; IQR: 4.9, 5.3) 18 months (median: 4.5; IQR: 3.9, 5.0). Milk LNS baseline (median: 5.2; IQR: 5.0, 5.4) 18 months (median: 4.7; IQR: 4.3, 4.8). Soya LNS baseline (median: 5.2; IQR: 5.0, 5.4) 18 months (median: 4.7; IQR: 4.2, 5.0). **Lactobacillus** log-transformed normalised read counts CBS: baseline (median: 3.8; IQR: 2.4, 4.1) 18 months (median: 3.5; IQR: 2.6, 4.1). Milk LNS baseline (median: 3.7; IQR: 2.7, 4.4) 18 months (median: 3.7; IQR: 3.3, 4.0). Soya LNS baseline (median: 3.6; IQR: 2.8, 4.1) 18 months (median: 3.9; IQR: 3.2, 4.3).	A small fraction of samples were found. **Salmonella**-positive: CSB: 8.1% at baseline, and 3.2% at 18 months. Milk LNS: positive 0% at baseline, and 8.0% at 18 months. Soya LNS: positive: 3.6% at baseline, and 3.6% at 18 months. **Shigella** log-transformed normalised read counts CBS: baseline (median: 2.0; IQR: 1.5, 2.5) 18 months (median: 0.7; IQR: 0.0, 1.4). Milk LNS baseline (median: 2.1; IQR: 1.6, 2.6) 18 months (median: 1.0; IQR: 0.0, 1.7). Soya LNS baseline (median: 2.0; IQR: 1.4, 2.7) 18 months (median: 0.5; IQR: 0.0, 1.1). **Escherichia** log-transformed normalised read counts CBS: baseline (median: 1.2; IQR: 0.0, 1.7) 18 months (median: 0.0; IQR: 0.0, 0.8). Milk LNS baseline (median: 1.2; IQR: 0.0, 1.8) 18 months (median: 0.0; IQR: 0.0, 0.8). Soya LNS baseline (median: 1.4; IQR: 0.0, 1.9) 18 months (median: 0.0; IQR: 0.0, 0.0).	46	**Bifidobacteria** decreased over time (each *p* < 0.001). **Lactobacilli:** relatively constant. **Bifidobacterium** log-transformed normalised read counts. Control: baseline (median: 5.1; IQR: 5.0, 5.4) 18 months (median: 4.7; IQR: 4.3, 5.0). Lactobacillus log-transformed normalised read counts. Control: baseline (median: 3.6; IQR: 2.6, 4.4) 18 months (median: 3.7; IQR: 3.3, 4.0).	A small fraction of samples were found. **Salmonella**-positive: 6.5% controls at baseline, and 10.9% control at 18 months. **Shigella** log-transformed normalised read counts. Control: baseline (median: 2.5; IQR: 1.9, 3.0) 18 months (median: 0.8; IQR: 0.0, 1.6). **Escherichia** log-transformed normalised read counts. Control: baseline (median: 1.5; IQR: 1.0, 2.1) 18 months (median: 0.0; IQR: 0.0, 0.8).
**Tang** [10]	2017	Kenya	Double-blind RCT	Iron fortification with MNPs.	6 months	3 months	13	Decrease in the relative abundance of **bifidobacterium** in MNP + Fe group (−6.38 +/− 2.5%, *p* = 0.02).	No significant decrease in the relative abundance of **Escherichia/Shigella** in the MNP + Fe group (−6.0 +/− 9%, *p* = 0.41).	20	No decrease in the relative abundance of **bifidobacterium** in the MNP-Fe group (−4.3 +/− 5%, *p* = 0.44). Decrease in the relative abundance of **bifidobacterium** in control group (−8.05 +/− 1.46%, *p* = 0.01).	Significant decrease in the relative abundance of **Escherichia/Shigella** in the MNP-Fe group (−16.05 +/− 6.9%, *p* = 0.05). **Clostridium** increased abundance in MNP-Fe only (1.94 +/− 2%, *p* = 0.007). Significant decrease in the relative abundance of **Escherichia/Shigella** in the control group (−19.75 +/− 4.5%, *p* = 0.01).
**Qasem** [44]	2017	Canada	RCT	Iron-fortified cereal.	4–6 months	2–4 weeks	37	Median relative abundance of **bifidobacteriaceae:** before CF: in Fe-cereal 50.016, in Fe + fruit: 58.638; after CF: in Fe-cereal 37.257, in Fe + fruit: 50.446. Median relative abundance of **lactobacillales:** before CF: in Fe-cereal 0.008, in Fe + fruit: 0.013; after CF: in Fe-cereal 0.014, in Fe + fruit: 0.033.	Median relative abundance of **bacteroidetes:** before CF: in Fe-cereal 4.789, in Fe + fruit: 0.112; after CF: in Fe-cereal 13.511, in Fe + fruit: 5.993. Median relative abundance of **enterobacteriaceae:** before CF: in Fe-cereal 6.544, in Fe + Fruit: 3.697; after CF: in Fe-cereal 4.894, in Fe + fruit: 6.617. Median relative abundance of **enterococcaceae:** before CF: in Fe-cereal 0.400, in Fe + fruit: 2.079; after CF: in Fe-cereal 1.152, in Fe + fruit: 1.326. Median relative abundance of **clostridia:** before CF: in Fe-cereal 0.003, in Fe + fruit: 0.006; after CF: in Fe-cereal 0.018, in Fe + fruit: 0.003.	19	Median relative abundance of **bifidobacteriaceae:** before CF: in meat group 41.377; after CF: in meat group 41.725. Median relative abundance of **lactobacillales** in meat group: before CF: 0.026; after CF: 0.026	Median relative abundance of **bacteroidetes** in meat group: before CF: 0.031; after CF: 2.246. Median relative abundance of **enterobacteriaceae** in meat group: before CF: 7.836; after CF: 10.330. Median relative abundance of **enterococcaceae** in meat group: before CF: 1.041; after CF: 2.084. Median relative abundance of **clostridia:** before CF: in meat group 0.003; after CF: 0.026
**Simonyte Sjodin** [45]	2019	Sweden	RCT	Iron-fortified milk and iron supplementation.	6 months	45 days	35	High iron formula: decreased relative abundance of **bifidobacterium** (*p* < 0.001, 60% vs. 78%) after only 45 days of intervention. High iron formula: relative abundance of **lactobacillus** sp (42%). Iron drops group: relative abundance of **lactobacillus** sp (8%).	High iron formula: no enhanced growth of **pathogenic** bacteria. High iron formula: abundance of **streptococcus** (0.9%), **clostridium** (9%), and **bacteroides** (0.9%). Iron drops group: abundance of **streptococcus** (0.2%), **clostridium,** (25%) and **bacteroides** (1.2%).	18	Relative abundance of **lactobacillus** sp (32%); *In this study, all groups received formula with added galacto-oligosaccharides (GOS) at 3.3 g/L.	No data

N1: number of cases fed with iron supplement; N2: number of cases not taking iron supplement.

## Data Availability

The data presented in this study are openly available in the relevant published articles.

## Supplementary Materials

The following supporting information can be downloaded at https://www.mdpi.com/article/10.3390/children11020231/s1. Table S1: Data on the microbiome from included studies; Table S2: PRISMA_2020_checklist.
