# Supplementary material for: The Effect of Oral Iron Supplementation/Fortification on the Gut Microbiota in Infancy: A Systematic Review and Meta-Analysis
Source: Children (Basel). 2024 Feb 10;11(2):231. doi: 10.3390/children11020231 (PMC10887499; doi:10.3390/children11020231)
Supplement: Supplementary file 1 [file children-11-00231-s001.zip › children-2853775-supplementary.pdf]

Table S1. Data on the microbiome from included studies

First Author	Year	Nutrition	Group	Participants	Species	Before	After	Change	Change SD
Tang	2017	MNP+Fe	Iron	13	Bifidobacteria	17.3±11.1%	-6.38±2.5%, p = 0.02	-6.38%	2.50%
Tang	2017	MNP+Fe	Iron	13	Escherichia	30.1%±23%	-6.23±9%, p = 0.41	-6.23%	9.00%
Tang	2017	MNP only	No iron	13	Bifidobacteria	17.3±11.1%	-4.27±5%, p = 0.4445	-4.27%	5.00%
Tang	2017	MNP only	No iron	13	Escherichia	30.1%±23%	-16.05±6.9, p = 0.05	-16.05%	6.90%
Tang	2017	Placebo	No iron	7	Bifidobacteria	17.3±11.1%	-8.05±1.46, p = 0.01	-8.05%	1.46%
Tang	2017	Placebo	No iron	7	Escherichia	30.1%±23%	-19.75±4.5%, p = 0.01	-19.75%	4.50%
Sjödin	2019	High iron	Iron	18	Bifidobacteria	78%	60.00%	-18%	17.75%
Sjödin	2019	High iron	Iron	18	Lactobacillus		42.00%		
Sjödin	2019	High iron	Iron	18	Streptococcus		0.90%		
Sjödin	2019	High iron	Iron	18	Clostridium		9.00%		
Sjödin	2019	High iron	Iron	18	Bacteroides		0.90%		
Sjödin	2019	Low iron	Iron	18	Bifidobacteria		78.00%		
Sjödin	2019	Low iron	Iron	18	Lactobacillus		32.00%		
Sjödin	2019	Iron drops	Iron	17	Bifidobacteria		N/A		
Sjödin	2019	Iron drops	Iron	17	Lactobacillus		8.00%		
Sjödin	2019	Iron drops	Iron	17	Streptococcus		0.20%		
Sjödin	2019	Iron drops	Iron	17	Clostridium		25.00%		
Sjödin	2019	Iron drops	Iron	17	Bacteroides		1.20%		
Krebs	2013	Fe	Iron	4	Bifidobacteria			-15%	34.99%
Krebs	2013	Fe	Iron	4	Lactobacillus			-15%	34.99%
Krebs	2013	Fe+Zn		6	Bifidobacteria			0%	0.80%
Krebs	2013	Fe+Zn		6	Lactobacillus			0%	0.80%
Krebs	2013	Meat	No iron	4	Bifidobacteria			0%	1.00%
Krebs	2013	Meat	No iron	4	Lactobacillus			0%	1.00%
Qasem	2017	Fe	Iron	18	Bifidobacteria	50%	37.30%	-13%	15.54%
Qasem	2017	Fe	Iron	18	Lactobacillus	0.20%	0.50%	0%	0.46%
Qasem	2017	Fe + fruits	Iron	19	Bifidobacteria	58.60%	50.40%	-8%	12.20%
Qasem	2017	Fe + fruits	Iron	19	Lactobacillus	0.50%	0.40%	0%	0.45%
Qasem	2017	Meat	No iron	19	Bifidobacteria	41%	41.70%	1%	4.47%
Qasem	2017	Meat	No iron	19	Lactobacillus	0.20%	0.10%	0%	0.45%

SD: Standard Deviation, Before: measure of the species before intervention, After: measure of the species after intervention
